# Sublingual epidermoid cyst: a case report

**DOI:** 10.1186/1752-1947-1-87

**Published:** 2007-09-17

**Authors:** Tolga Kandogan, Murat Koç, Enver Vardar, Elif Selek, Özlem Sezgin

**Affiliations:** 1 Department of Otolaryngology, İzmir Teaching and Research Hospital, Bozyaka 35220 İzmir, Turkey; 2 Department of Pathology, İzmir Teaching and Research Hospital, Bozyaka 35220 İzmir, Turkey; 3 Department of Radiology, İzmir Teaching and Research Hospital, Bozyaka 35220 İzmir, Turkey

## Abstract

Epidermoid and dermoid cysts represent less than 0.01% of all oral cavity cysts. The cysts can be defined as epidermoid when the lining presents only epithelium, dermoid cysts when skin adnexa are found, and teratoid cysts when other tissue such as muscle, cartilage, and bone are present.

In this article, we present the case of an epidermoid cyst, with an oral as well as a submental component, in an 11 year old boy who presented with complaints of a mass in the oral cavity, difficulty chewing and swallowing of solid foods for about 3 years. He was admitted to the otolaryngology department. On examination, a mass displacing the tongue superiorly and posteriorly was noticed. An MRI scan was done and showed a 40 × 35 mm well-circumscribed non-enhancing cystic mass extending from the sublingual area to the level of the thyroid notch. The content of the cyst was homogenous. On examining the neck, a firm swelling was also noticed in the submental area, extending down to the thyroid notch. Under general anesthesia and with nasotracheal intubation, the patient underwent surgical removal of the mass. Extraorally, a midline submental horizontal incision was performed through the mucosa overlying the swelling and the cyst was dissected from the surrounding tissues and removed. On histological examination, acidophilic stratum corneum and basophilic dot like staining of stratum granulosum, which is the hallmark of an epidermoid cyst, were seen. The patient did well postoperatively, and no recurrence was noticed at the 6-months follow-up.

## Introduction

Epidermoid and dermoid cysts are benign lesions encountered throughout the body, with 7% occurring in the head and neck area and 1.6% within the oral cavity [1]. They represent less than 0.01% of all oral cavity cysts [2]. The cysts can be defined as epidermoid when the lining presents only epithelium, dermoid cysts when skin adnexa are found, and teratoid cysts when other tissue such as muscle, cartilage, and bone are present [3].

The pathogenesis of midline cysts of the floor of the mouth is not well established, and dysontogenetic, traumatic, and thyroglossal anomaly theories have been suggested. Histologically, Meyer divided the cysts of the floor of the mouth into three groups: epidermoid, dermoid, and teratoid [4]. Although dermoid cysts represent a separate entity, the term "dermoid" is typically used to indicate all three categories [4]. In fact, dermoid cysts occur primarily in the testes and ovaries, and the most common location in the head and neck is the external third of the eyebrow [4].

Dermoid cysts generally present with slow and progressive growth, and even if they are congenital, the diagnosis is usually possible in the second or third decade of life [5].

The treatment of dermoid cysts of the floor of the mouth is surgical and can be by an intraoral or extraoral route according to the localization and the size of the mass [6].

Dermoid cysts usually present early in life as an asymptomatic mass and are treated by simple excision. However, they may reach a large size, involve more than one anatomical area and/or abut the hyoid bone when in the neck [7].

Such a swelling on the floor of the mouth can occasionally cause serious problems for swallowing and speaking [8,9].

In this article, we outline the case of an epidermoid cyst with an oral as well as a submental component diagnosed in an 11 year old boy.

## Case presentation

An 11 year old male patient was admitted to the otolaryngology department with complaints of a mass in the oral cavity and difficulty chewing and swallowing of solid foods for the past 3 years. The patient had no dyspnea or pain. There was no history of previous surgery or trauma to the oral cavity or neck. On examination, there was a 40 × 35 mm sublingual mass with normal covering mucosa displacing the tongue superiorly and posteriorly.

**Figure 1:**
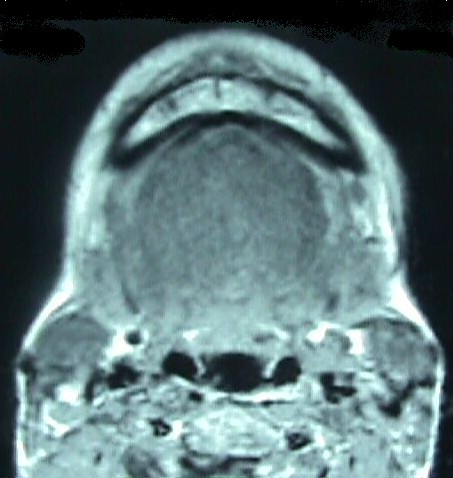
A MRI scan showing an 40 × 35 mm well-circumscribed non-enhancing cystic mass extending from the sublingual area to the thyroid notch level.

On examining the neck, a firm swelling was also noticed in the submental area, extending down to the thyroid notch. An MRI scan was done and showed an 40 × 35 mm well-circumscribed non-enhancing cystic mass extending from the sublingual area to the thyroid notch level (Figure 1). The content of the cyst was homogenous. In order to exclude any thyroid pathology, thyroid scintigraphy was taken. This was also normal.

**Figure 2:**
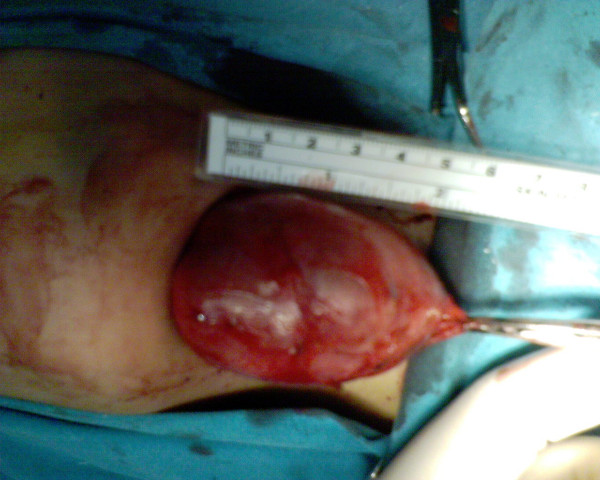
A per-operative view to the cyst.

**Figure 3:**
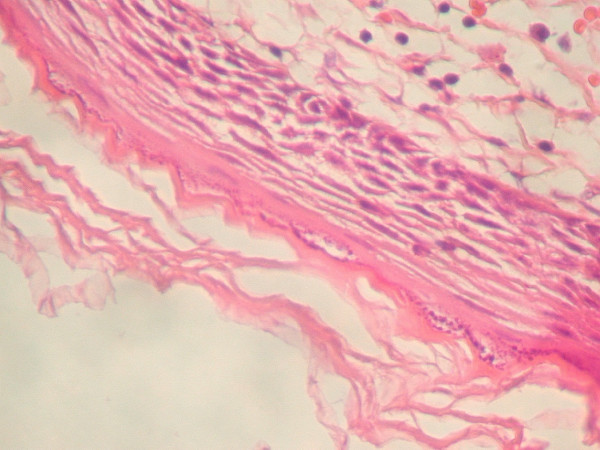
Acidophilic stratum corneum and basophilic dot like staining of stratum granulosum were seen. Stratum granulosum is hallmark of epidermoid cyst. (H-E –200).

Under general anesthesia and with nasotracheal intubation, the patient underwent surgical removal of the mass. Extraorally, a midline submental horizontal incision was performed through the mucosa overlying the swelling and the cyst was dissected from the surrounding tissues and removed (Figure 2). The wound was closed primarily. The postoperative period was without any complication and the tongue went back to its normal position. On histological examination, acidophilic stratum corneum and basophilic dot like staining of stratum granulosum were seen (Figure 3). Stratum granulosum is the hallmark of epidermoid cyst. (H-E ×200). It confirmed the diagnosis of an epidermoid cyst. The patient did well postoperatively, and no recurrence was noticed at the 6-months follow-up.

## Discussion

Epidermoid cysts may be classified as congenital or acquired, even if there is no difference between the two on presentation or histologically. Many etiopathogenetical theories have been proposed. Congenital cysts are dysembryogenetic lesions that arise from ectodermic elements entrapped during the midline fusion of the first and second branchial arches between the third and fourth weeks of intrauterine life. Alternatively, they may arise from the tuberculum impar of His which, with each mandibular arch, forms the floor of the mouth and the body of the tongue. Acquired cysts derive from traumatic or iatrogenic inclusion of epithelial cells or from the occlusion of a sebaceous gland duct. Moreover, others authors proposed that midline cysts may represent a variant form of thyroglossal duct cyst [4,6,8,10].

Congenital cysts of ectodermal origin are uncommon in the oral cavity (1.6%), with epidermoid cysts rarely occurring there [11]. Midline cysts of the floor of the mouth are painless lesions that swell from the anterior portion of this region. Because they can displace the tongue, patients usually present with dysphagia, dysphonia, and dyspnea, and in the case of lower localization, they present a characteristic double chin [6].

Dermoid cysts are generally diagnosed in young adults in the second and third decades of life [6]; although the case presented here was an 11 year old boy.

There are no rules regarding the timing for operation; because dermoid cysts are mainly congenital, they can appear in every age of life, so the time when they appear (generally with dysphagia, dysphonia, and dyspnea) is generally the right time to operate on them. Also, in very young patients, a problem can arise from the anesthesiologic risk, which is generally quite low in patients weighing more than 20 kg [6].

Histologically, midline dermoid cysts of the floor of the mouth are classified according to Meyer's classification, thus dividing them into three groups: epidermoid cysts, which consist of an epithelial-lined wall that may be partly keratinized; dermoid cysts, which are epidermoid-like cysts but show evidence of skin appendages, such as hair follicles, hair, sweat, and sebaceous glands; and teratomas, which contain, in addition to skin appendages, mesodermal elements such as bone, muscle, respiratory and gastrointestinal tissues, and a fibrous capsule. The latter type is the only variety that may have a malignant change [3,6,8,12].

Anatomic classification divides the cysts of the floor of the mouth into three groups according to their relation to the muscles of the floor of the mouth : sublingual or median genioglossal cysts, located above the geniohyoid muscles; median geniohyoid cysts, located in the submental region between the geniohyoid and mylohyoid muscles; and lateral cysts, located in the submaxillary region [6].

The differential diagnosis of sublingual lesions includes: infectious process, ranula, lymphatic malformation, dermoid cyst, epidermoid cyst, heterotopic gastrointestinal cyst and duplication foregut cyst. For this reason, bimanual palpation and conventional radiography are not always sufficient in making differential diagnoses. In these cases, it is necessary to use ultrasonography, computed tomography, or magnetic resonance imaging together with cytologic examination by fine-needle aspiration biopsy [8]. Ultrasonography represents the first choice of imaging technique because it is reliable, economical, and without x-ray exposure, so it is easily suitable for young patients also. Computed tomography and magnetic resonance imaging allow more precise localization of the lesion in relationship to geniohyoid and mylohyoid muscles, and they also enable the surgeon to choose the most appropriate surgical approach, especially for very large lesions [6].

Surgical enucleation is the only effective treatment for these kinds of lesions. Several techniques are reported in the literature, which may be divided into intraoral and extraoral techniques depending on which approach is used [6]. The extraoral approach is generally preferred in the case of median geniohyoid or very large sublingual cysts, whereas the intraoral approach is typically used for smaller sublingual cysts [13].

Prognosis is very good, with a very low incidence of relapse, usually related to the genial tubercles or to the hyoid bone. Malignant changes have been recorded in dermoid cysts by New and Erich but not in the floor of the mouth, although a 5% rate of malignant transformation of oral dermoid cysts of the teratoid type has been reported by other authors [5].

## Conclusion

Appropriate imaging techniques and thyroid scintigraphy are necessary in the preoperative diagnosis of cysts of the floor of the mouth. Surgical enucleation is the only effective treatment for these kinds of lesions.

## Competing interests

The author(s) declare that they have no competing interests.

## Authors' contributions

TK, MK, EV, ES and ÖS drafted the manuscript and designed the case report. All authors read and approved the final manuscript.
